# Patterns of symptoms before a diagnosis of first episode psychosis: a latent class analysis of UK primary care electronic health records

**DOI:** 10.1186/s12916-019-1462-y

**Published:** 2019-12-04

**Authors:** Ying Chen, Saeed Farooq, John Edwards, Carolyn A. Chew-Graham, David Shiers, Martin Frisher, Richard Hayward, Athula Sumathipala, Kelvin P. Jordan

**Affiliations:** 1 School of Primary, Community and Social Care, Keele University, Keele, ST5 5BG UK; 2 University of Manchester, Manchester, M13 9PL UK; 3 Psychosis Research Unit, Greater Manchester Mental Health NHS Trust, Manchester, M25 3BL UK; 4 School of Pharmacy, Keele University, Keele, ST5 5BG UK

**Keywords:** First episode psychosis, Symptom cluster, General practice, Medical record research, Latent class analysis, Epidemiology

## Abstract

**Background:**

The nature of symptoms in the prodromal period of first episode psychosis (FEP) remains unclear. The objective was to determine the patterns of symptoms recorded in primary care in the 5 years before FEP diagnosis.

**Methods:**

The study was set within 568 practices contributing to a UK primary care health record database (Clinical Practice Research Datalink). Patients aged 16–45 years with a first coded record of FEP, and no antipsychotic prescription more than 1 year prior to FEP diagnosis (*n* = 3045) was age, gender, and practice matched to controls without FEP (*n* = 12,180). Fifty-five symptoms recorded in primary care in the previous 5 years, categorised into 8 groups (mood-related, ‘neurotic’, behavioural change, volition change, cognitive change, perceptual problem, substance misuse, physical symptoms), were compared between cases and controls. Common patterns of symptoms prior to FEP diagnosis were identified using latent class analysis.

**Results:**

Median age at diagnosis was 30 years, 63% were male. Non-affective psychosis (67%) was the most common diagnosis. Mood-related, ‘neurotic’, and physical symptoms were frequently recorded (> 30% of patients) before diagnosis, and behavioural change, volition change, and substance misuse were also common (> 10%). Prevalence of all symptom groups was higher in FEP patients than in controls (adjusted odds ratios 1.33–112). Median time from the first recorded symptom to FEP diagnosis was 2–2.5 years except for perceptual problem (70 days). The optimal latent class model applied to FEP patients determined three distinct patient clusters: ‘no or minimal symptom cluster’ (49%) had no or few symptoms recorded; ‘affective symptom cluster’ (40%) mainly had mood-related and ‘neurotic’ symptoms; and ‘multiple symptom cluster’ (11%) consulted for three or more symptom groups before diagnosis. The multiple symptom cluster was more likely to have drug-induced psychosis, female, obese, and have a higher morbidity burden. Affective and multiple symptom clusters showed a good discriminative ability (C-statistic 0.766; sensitivity 51.2% and specificity 86.7%) for FEP, and many patients in these clusters had consulted for their symptoms several years before FEP diagnosis.

**Conclusions:**

Distinctive patterns of prodromal symptoms may help alert general practitioners to those developing psychosis, facilitating earlier identification and referral to specialist care, thereby avoiding potentially detrimental treatment delay.

## Background

There is often a substantial gap between the first presentation of symptoms and subsequent diagnosis of a first episode psychosis (FEP) leading to a delay in treatment and worse outcomes [1]. The average duration of untreated psychosis (DUP), the period between the first onset of psychotic symptoms and treatment, has been reported to be over a year [2–4]. Several independent meta-analyses have provided evidence for the association between long DUP and poor outcome. Marshall et al. included 26 studies involving prospective cohorts with over 4000 participants. This meta-analysis revealed significant associations between long DUP and poor outcomes in symptomatic and functional domains at 6 and 12 months after diagnosis, which were independent of co-morbidity [5]. Perkins et al. included 44 studies involving 5491 participants and demonstrated that longer DUP was associated with less response to antipsychotic medication [1]. Penttilä et al. included 33 studies with a mean follow-up of 8.1 years and concluded that long DUP was associated with poor general symptomatic outcome, more severe positive and negative symptoms, lesser likelihood of remission, and poor social functioning and global outcome in the long term [4]. Reducing DUP has become the primary aim of modern psychiatric services for patients with a FEP.

Initiatives to shorten DUP, however, have largely been unsuccessful [6]. This is partly due to the fact that the nature of symptoms in the prodromal period remains unclear. In a systematic review, Anderson et al. explored the nature of the pathway to care for patients experiencing a FEP. Almost all studies in this review explored the sex, socio-economic, or ethnic determinants of the pathways to care. The authors commented that the nature of the pathway to care, which includes understanding the symptoms and presentations in the period before the diagnosis of FEP, is crucial in understanding the delay in contact with services [7]. Some studies have examined the help-seeking behaviour in terms of psychological processes such as locus of control or type of symptoms leading to contacts with health professionals before FEP diagnosis [8, 9]. Platz et al. found that patients with psychotic symptoms more often contacted mental health professionals, whereas patients with insidious and more unspecific features more frequently contacted general practitioners (GPs) [9]. Anderson et al. also found in their review that in 13 of 21 studies which examined pathways to care, the first contact for most patients was with a physician (including all 3 UK studies). However, the referral source to psychiatric services for the greatest proportion of patients was emergency services in 9 of 22 studies which examined such topic (including 2 of 4 UK studies) [7]. In addition, data from the UK suggested that individuals referred by a home treatment team or the emergency service had the lowest DUP [10]. These probably reflected the lack of identification of the symptoms suggestive of FEP in routine care settings, including primary care services. This is despite the evidence that contacts with primary care may increase prior to the diagnosis of FEP: a Danish register-based study suggested an increase in primary care contacts several years prior to the diagnosis of schizophrenia [11]. A UK study also demonstrated that in general, higher frequency of GP contact before the onset of psychosis was associated with shorter DUP [8]. However, a small study in Switzerland found that patients consulted GPs with insidious features which were not recognised by GPs as being an indication of FEP, hence causing delays in referral to specialised services, a diagnosis made, and treatment initiated [9].

Patterns of consultation and presentation of symptoms in primary care before the diagnosis of psychosis have not been fully studied. Identification of these symptoms, including psychological and physical and substance misuse, may help to identify patients earlier, thus helping to reduce unacceptably long DUP. The objective of this study was to determine common symptoms and patterns of symptomatology presented to primary care prior to diagnosis of FEP. This would help inform the need for better and more targeted risk management by clinicians when patients present with symptoms suggestive of FEP.

## Methods

### Setting

The study was set within the Clinical Practice Research Datalink (CPRD). CPRD is a pseudo-anonymised database of routinely recorded general practice information from over 10 million patients registered with over 670 UK primary care practices [12]. Diagnosis of mental and behavioural disorders recorded in CPRD has been validated using internal (i.e. manual review of diagnostic algorithm) and external (i.e. questionnaire to GP, record request to GP, questionnaire and record request to GP, comparison of rates) methods by 20 studies [13]. It was also shown that the rates of GP recorded severe mental illness in UK primary care were broadly comparable to incidence rates from previous epidemiological studies of severe mental illness in the UK [14].

### Study population

We included patients aged 16–45 years with a first coded record of a FEP (defined as affective, non-affective, drug-induced, and pregnancy-related psychoses) between 1 April 2005 (1 year after the introduction of the Quality and Outcomes Framework (QOF) into UK primary care [15]) and 31 December 2016, who had been registered at a practice contributing to CPRD for at least 5 years before the recorded FEP, and had not received an antipsychotic prescription more than 1 year prior to FEP. The rationale for the antipsychotic prescription criterion was that treatment may commence before a formal diagnosis has been entered onto the clinical system, but if treatment is recorded more than a year before diagnosis, this would suggest ongoing active treatment but inadequate diagnostic recording in primary care. Patients with a recorded code for Parkinson’s disease or dementia in the 5 years prior to FEP were ineligible. Patients with a coded record of psychosis in remission during the 5-year period were also excluded.

A control group was identified, matched 4:1 [16] by year of birth (age), gender, and practice, to the FEP group. The controls had no recorded consultations for FEP, psychosis in remission or antipsychotic prescription, and had at least 5 years prior registration before the index date (which was the diagnosis date of FEP for their matched case).

### Exposure

Recorded symptoms potentially related to FEP in the 5 years prior to the index date in both patients with FEP and controls were identified and categorised into eight groups. These symptom groups were determined through a review of the literature [17–19] and consensus of a consultant in psychiatry (SF) and two general practitioners with electronic health records research experience (JE, RH). In general, the final eight groups were based on the concept of Yung and McGorry as a foundation [17] and included psychological, substance-related, and physical symptoms (Table 1).

**Table 1:** Groups of prodromal symptoms in psychosis

Symptom group	Individual symptoms^†^ included in each group
Psychological symptom	
Mood-related symptom	Depression, anhedonia, guilt, mood swings, suicidal/self-harm ideation or behaviour
‘Neurotic’ symptom	Neuroses, anxiety, irritability and anger, restlessness, worrying thoughts
Behavioural change	Deterioration, social withdrawal, impulsivity, reduced self-esteem, aggressive and disruptive behaviour, odd behaviour
Change in volition	Apathy (loss of drive), tiredness/fatigue (loss of energy), boredom (loss of interest)
Perceptual problem	Hallucinations, delusions, illusions
Cognitive change	Disturbance of attention, concentration/preoccupation difficulties, cognitive/memory impairment, thought disorder/blocking
Substance misuse	Opioids, alcohol, cannabis, hypnotic, cocaine, amphetamine, glue, tobacco, hallucinogen, ecstasy, antidepressant, solvent, other/multiple stimulant, general (codes without specific substance)
Physical symptom	Speech abnormality, sleep disturbance, loss of weight, poor appetite, dryness of the mouth, dysphagia, hyperventilation, muscle tension, epigastric discomfort, palpitations, shortness of breath, excessive wind, decreased libido, menstrual problem (in females), failure of erection (in males)

^†^Total number of individual symptoms studied, *n* = 55

### Covariates

Covariates thought to be potentially associated with symptoms/psychosis and recorded in CPRD were year of the index date, age, gender, geographical region, smoking status, alcohol consumption, body mass index (BMI), specific co-morbidities, total morbidity burden, and the frequency of GP consultation.

At the practice level, the geographical region was recorded by CPRD as 1 of 13 regions in the UK [12]. In this study, these regions were further summarised into London, South England (South West, South Central, South East Coast), Midlands and East England (East Midlands, West Midlands, East of England), North England (North East, North West, Yorkshire, and the Humber), Wales, Scotland, and Northern Ireland.

Smoking and alcohol information was classified as ever smoked/drunk alcohol, never, or missing, based upon the data recorded before the index date. The BMI value used was the most recent record before the index date and was grouped into < 18.5 (underweight), ≥ 18.5 and < 25 (normal), ≥ 25 and < 30 (overweight), ≥ 30 kg/m^2^ (obese), or missing. Sensitivity analyses in patients with and without missing data on smoking, alcohol drinking, and BMI are briefly shown in Additional file 1. Findings were similar in both groups.

Physical comorbidities often co-exist in people with a mental health problem [20]. Common conditions previously found to be associated with psychosis were included in this study. Candidate conditions included diabetes, ischaemic heart disease, asthma, chronic obstructive pulmonary disease, inflammatory diseases (rheumatoid arthritis, gout, polymyalgia rheumatic, inflammatory bowel disease, systemic lupus erythematosus, spondyloarthritis), hypertension, chronic kidney disease, musculoskeletal pain (back, foot/ankle, hand/wrist, hip, knee, neck, shoulder), and injury and major trauma. These conditions were identified during the 5-year period prior to the index date.

In addition, prescriptions for drugs recorded under different British National Formulary (BNF) chapters in the 5-year period prior to the index date was used as a surrogate measure of the total morbidity burden, which has been shown to be as predictive of health outcomes as more complicated comorbidity measures [21].

The frequency of GP consultation was determined annually over the 5 years prior to the index date.

### Codes and identification

In UK primary care, problems, including symptoms and diagnoses, are generally recorded using the ‘Read’ system of codes [22]. Diagnosis of FEP and prodrome symptoms were identified by the use of a Read code list developed through consensus of clinical researchers (SF, JE, and RH, see Additional file 2). Identification of co-morbid conditions used prior established Read code lists within the research institute.

Antipsychotic medications were defined as medications under BNF chapter 4.2 (by JE, see Additional file 2).

### Statistical analysis

The recorded consultation prevalences of each individual symptom and symptom group (at least one recorded symptom from a group) in patients with FEP and matched controls during the 5-year prior to the index date were determined. Conditional logistic regression was used to analyse the associations of individual symptoms and symptom groups with FEP in separate models, adjusted for the other covariates and reported using odds ratios (ORs) with 95% confidence intervals (CIs). Cluster-robust variance estimators were used to take into account clustering by practice.

For patients with FEP, for each symptom group, the time interval between the first recorded symptom (if any) and diagnosis was determined. We also identified the earliest record (if any) of symptoms, regardless of symptom group, for each patient.

Within patients with FEP, latent class analysis (LCA) was used to determine common patterns of symptoms, at the level of the symptom groups, presented to primary care over the 5-year period before FEP diagnosis. The LCA clustered patients with FEP into distinct groups based on their pattern of prior recorded symptoms across the 8 symptom groups, with each patient allocated to one cluster [23]. We used *L*^2^ statistics with bootstrap *p* values, Bayes Information Criterion (BIC), and Consistent Akaike’s Information Criterion (CAIC) to determine the optimal model, i.e. the optimal number of clusters. Latent GOLD (version 4.5) was used to perform the analyses, using both the estimation-maximisation and Newton-Raphson algorithms to estimate model parameters. One thousand different random starting values were used, each of which included 100 iterations. Bootstrap *p* values based on 500 replications were determined to assess the model fit based on the *L*^2^ statistics. The optimal number of clusters is where the *p* value becomes non-significant at the desired significance level (5%). For the BIC and CAIC, the optimal model is the model with the smallest information criterion values. Patients were allocated to clusters based on their posterior probabilities of belonging to each cluster. A mean posterior probability ≥ 0.7 for patients allocated to a cluster was considered acceptable [24]. Based on the optimal model, the identified clusters were compared at diagnosis on patient characteristics (psychosis subtype, age, gender, geographical region, smoking, alcohol drinking, BMI, morbidity burden, frequency of consultation). The proportions of patients in each cluster with pre-recorded symptoms 4, 3, 2, and 1 year before diagnosis were identified.

We also mapped each control to the clusters that had been identified in cases based on their recorded symptoms during the 5 years before the index date. Then, in the case-control setting, a conditional logistic regression model containing symptom cluster as an independent variable was used to determine the discriminative ability of these clusters to predict a FEP diagnosis, assessed using C-statistic with 95% CIs. A value ≥ 0.75 was considered to indicate good discrimination.

All analyses were performed using STATA/MP 15 if not stated elsewhere.

### Patient and public involvement

The research findings were discussed with a convenor of a local support group (‘Hear Our Voices’, North Staffordshire, England) that aims to give a voice to people with a mental health problem.

## Results

### Patient characteristics

Three thousand forty-five FEP patients (63% male, median age 30) and 12,180 age-, gender-, and practice-matched controls were included in the analysis from 568 general practices. FEP patients were diagnosed most commonly with non-affective psychosis (67%), followed by drug-induced (22%), affective (10%), and pregnancy-related (1%) psychoses (Table 2).

**Table 2:** Participant demographic and clinical characteristics at the index date

	FEP patients (*n* = 3045)	Matched participants (*n* = 12,180)	*p* value
Type of psychosis, *n* (%)			
Non-affective	2036 (66.9)	NA	
Drug-induced	678 (22.3)	NA	
Affective	309 (10.2)	NA	
Pregnancy-related	22 (0.7)	NA	
Year of the index date, median (IQR)	2010 (2007, 2013)	2010 (2007, 2013)	Matched
Age, median (IQR)	30 (23, 39)	30 (23, 39)	Matched
Male, *n* (%)	1914 (62.9)	7656 (62.9)	Matched
Geographical region, *n* (%)			Matched
London	271 (8.9)	1084 (8.9)	
South England	798 (26.2)	3192 (26.2)	
Midlands and East England	599 (19.7)	2396 (19.7)	
North England	532 (17.5)	2128 (17.5)	
Northern Ireland	168 (5.5)	672 (5.5)	
Scotland	356 (11.7)	1424 (11.7)	
Wales	321 (10.5)	1284 (10.5)	
Smoking, *n* (%)			< 0.0001
Non-smoker	1011 (33.2)	6041 (49.6)	
Ever smoker	1764 (57.9)	4031 (33.1)	
Unknown	270 (8.9)	2108 (17.3)	
Alcohol consumption, *n* (%)			NS
Non-drinker	298 (9.8)	1026 (8.4)	
Ever drinker	1742 (57.2)	5907 (48.5)	
Unknown	1005 (33.0)	5247 (43.1)	
Body mass index, median (IQR)	23.8 (21.0, 27.9)	24.5 (21.6, 28.3)	< 0.0001
< 18.5 kg/m^2^ (underweight), *n* (%)	150 (4.9)	326 (2.7)	
≥ 18.5 kg/m^2^ and < 25 kg/m^2^ (normal), *n* (%)	1085 (35.6)	3578 (29.4)	
≥ 25 kg/m^2^ and < 30 kg/m^2^ (overweight), *n* (%)	501 (16.5)	2025 (16.6)	
≥ 30 kg/m^2^ (obese), *n* (%)	355 (11.7)	1355 (11.1)	
Unknown, *n* (%)	954 (31.3)	4896 (40.2)	
Number of different prescriptions in 5 years before the index date, median (IQR)	4 (3, 6)	3 (1, 5)	< 0.0001
Number of GP consultations in 5 years before the index date, median (IQR)	55 (29, 95)	25 (10, 53)	< 0.0001
4–5 years prior to the index date	6 (2, 15)^†^	3 (1, 9)	< 0.0001
3–4 years prior to the index date	7 (2, 16)^†^	4 (1, 10)	< 0.0001
2–3 years prior to the index date	8 (2, 18)^†^	4 (1, 11)	< 0.0001
1–2 years prior to the index date	9 (3, 20)^†^	4 (1, 11)	< 0.0001
0–1 year prior to the index date	17 (8, 30)^†^	4 (1, 12)	< 0.0001
Number of symptom records in 5 years before the index date, median (IQR)^‡^	2 (1, 5)	0 (0, 1)	< 0.0001

*p* value obtained from chi-squared or from Mann-Whitney *U* test as appropriate, and where applicable analysis excluded the ‘unknown’ category (missing data)

*FEP* first episode psychosis, *IQR* interquartile range, *NA* not applicable, *NS* not significant

^†^One-way ANOVA trend analysis within patients with FEP, *p* < 0.0001

^‡^Total number of coded records based on 55 studied individual symptoms

FEP patients were more likely than controls to smoke and had a lower BMI. Increased morbidity burden was observed in FEP patients (median of 4 different prescriptions in the previous 5 years versus 3 in the controls). The median number of GP consultations for FEP patients was more than double those of controls during the 5-year period before the index date. FEP patients increased from a median of 6 consultations in the time period 49–60 months before diagnosis to 17 consultations in the 12 months before diagnosis. The median number of recorded symptoms was 2 in FEP patients and 0 in controls over the 5 years (Table 2).

Respiratory comorbidity, musculoskeletal pain, and injury and major trauma were more common in FEP patients than in controls (Additional file 3).

### Prodrome symptoms

Prevalence of recorded individual symptoms over the 5 years is given in Additional file 4.

Prevalence of all symptom groups was higher in FEP patients than in controls (Table 3). In patients with FEP, 48% were recorded to have at least one mood-related symptom in the 5 years prior to FEP diagnosis, compared to 11% of controls (adjusted OR 7.6 (95% CI 6.8, 8.6)). ‘Neurotic’ symptoms, behavioural change, change in volition, and substance misuse were also frequently recorded (> 10%) in FEP patients. Perceptual problems, a typical psychotic symptom, were recorded in 5% of FEP patients but hardly in any controls. Physical symptoms were recorded in 31% of FEP patients and 16% of controls (adjusted OR 1.8; 95% CI 1.6, 2.0) (Table 3).

**Table 3:** Five-year prevalence of symptom groups in patients prior to psychosis and matched participants

Symptom group	FEP patients (*n* = 3045)		Matched participants (*n* = 12,180)		Crude odds ratio (95% CI)^‡^	Adjusted odds ratio^‡^* (95% CI)
	*n*^†^	5-year prevalence (%)	*n*^†^	5-year prevalence (%)		
Psychological symptom						
Mood-related symptom	1473	48.4	1275	10.5	10.1 (9.12, 11.3)	7.60 (6.75, 8.56)
‘Neurotic’ symptom	1133	37.2	1106	9.1	6.54 (5.89, 7.27)	4.87 (4.35, 5.47)
Behavioural change	490	16.1	657	5.4	3.53 (3.08, 4.05)	2.67 (2.30, 3.09)
Change in volition	394	12.9	923	7.6	1.88 (1.64, 2.16)	1.33 (1.14, 1.55)
Perceptual problem	162	5.3	5	0.04	130 (53.7, 313)	112 (44.4, 283)
Cognitive change	38	1.3	30	0.3	5.41 (3.35, 8.75)	3.80 (2.04, 7.09)
Substance misuse	338	11.1	134	1.1	13.0 (10.4, 16.4)	8.09 (6.36, 10.3)
Physical symptom	939	30.8	1926	15.8	2.70 (2.43, 2.99)	1.80 (1.61, 2.02)

*FEP* first episode psychosis, *CI* confidence interval

^†^Number of individuals with recorded symptom in the 5-year period before the index date

^‡^Conditional logistic regression analyses with cluster-robust variance estimator

*Adjusted for smoking, alcohol consumption, BMI morbidity burden, and specific co-morbid conditions (including respiratory condition, musculoskeletal pain, and injury and major trauma), in addition to matched year of birth (age), gender, and practice

### Time from the first symptom to diagnosis

Within each symptom group, the median time from first recorded symptom to diagnosis was around 2 to 2.5 years (range 732–975 days) although shorter for perceptual problems (70 days) (Additional file 5, Fig. 1a).

**Fig. 1:**
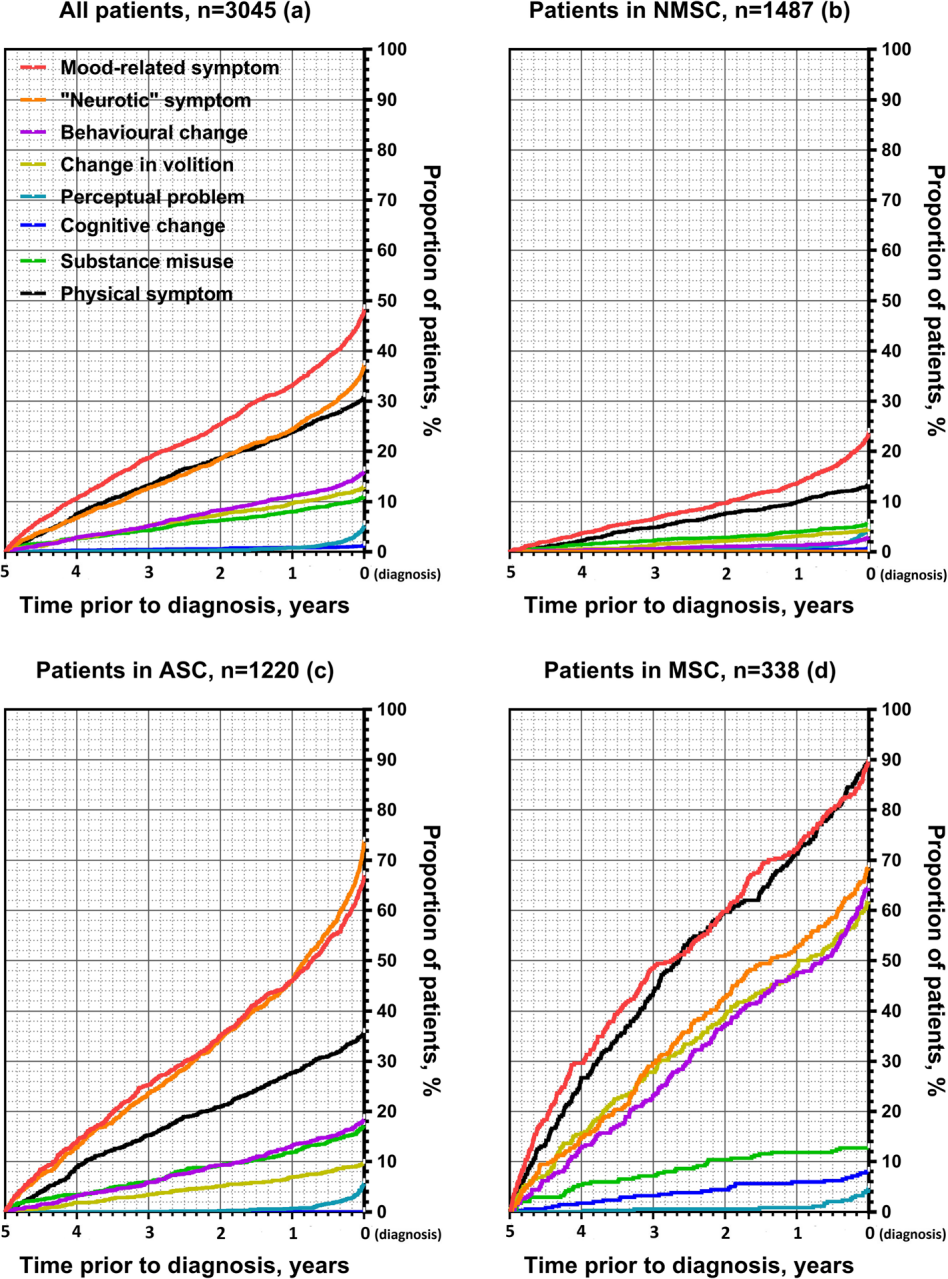
Cumulative proportion of patients having a prodrome symptom recorded in primary care from 5 years before until the time of FEP diagnosis. FEP, first episode psychosis; NMSC, no or minimal symptom cluster; ASC, affective symptom cluster; MSC, multiple symptom cluster

The earliest symptom, regardless of the symptom group, was recorded around 3 years (median 1065 days) before diagnosis (Additional file 6).

### Common symptom clusters in FEP patients

The LCA analysis using recorded symptom groups prior to the diagnosis of FEP resulted in the three-cluster model providing the best fit based on BIC, CAIC, and bootstrap *p* values (Additional file 7). FEP patients generally displayed high posterior probabilities for their assigned clusters, with mean posterior probabilities ranging from 0.71 to 0.81 across the three clusters (Table 4).

**Table 4:** Cluster classification: posterior probability of membership of clusters

Assigned cluster, *n* (%)	Mean posterior probability for each cluster		
	NMSC	ASC	MSC
NMSC, *n* = 1487 (48.8)	0.809	0.174	0.018
ASC, *n* = 1220 (40.1)	0.128	0.713	0.159
MSC, *n* = 338 (11.1)	0.022	0.236	0.743

*NMSC* no or minimal symptom cluster, *ASC* affective symptom cluster, *MSC* multiple symptom cluster

Based on the optimal model, 49% (*n* = 1487) of FEP patients were in a cluster characterised by no or minimal symptoms recorded in primary care in the 5 years before diagnosis (the ‘no or minimal symptom cluster’, Fig. 1b). Forty-nine per cent (734/1487) in this cluster had no prior recorded symptom, and 45% (669/1487) had presented with symptom(s) from only 1 symptom group, commonly a mood-related or physical symptom.

The second cluster contained 40% (*n* = 1220) of FEP patients, and these had consulted with symptoms from at least 1 symptom group (median number 2), and patients in the cluster mainly had ‘neurotic’ (74%) or mood-related (67%) symptoms (the ‘affective symptom cluster’, Fig. 1c). Forty-four per cent (541/1220) of patients in this cluster had consulted for both mood-related and ‘neurotic’ symptoms. All patients with a mood-related symptom in this cluster also presented with at least one other symptom from another symptom group.

Cluster 3 contained 11% (*n* = 338) of FEP patients, and they consulted across multiple symptom groups (median number 4) with high probability of consulting in particular for physical (90%), mood-related (90%), ‘neurotic’ (69%), behavioural (65%), and volition change (62%) symptoms (the ‘multiple symptom cluster’, Fig. 1d). All patients in cluster 3 consulted for symptoms in at least three symptom groups.

Relative to the affective symptom cluster, the no or minimal symptom cluster had a higher proportion of patients with non-affective psychosis, while the multiple symptom cluster included a higher proportion of patients with affective and drug-induced psychosis (Table 5). The no or minimal symptom cluster patients were the youngest (median age 29 years) and had the highest percentage being male (72%). The multiple symptom cluster patients were the oldest (median age 33 years) and had the lowest percentage being male (36%). This cluster also had the highest rates of recorded obesity, alcohol drinking, morbidity burden, and frequency of GP consultations (Table 5). Patients in the multiple symptom cluster had a median of 4 years (median 1548 days) from earliest symptom to diagnosis, with mood-related (31%) and physical (29%) as the most common earliest symptoms (Additional file 6).

**Table 5:** Characteristics at diagnosis of FEP patients by cluster

	Symptom clusters			*p* value^†^
	NMSC patients (*n* = 1487)	ASC patients (*n* = 1220)	MSC patients (*n* = 338)	
Type of psychosis, *n* (%)^‡^				< 0.0001
Non-affective	1023 (68.8)	819 (67.1)	194 (57.4)	
Drug-induced	305 (20.5)	277 (22.7)	96 (28.4)	
Affective	146 (9.8)	117 (9.6)	46 (13.6)	
Male, *n* (%)	1064 (71.6)	727 (59.6)	123 (36.4)	< 0.0001
Age, median (IQR)	29 (21, 38)	31 (24, 38)	33 (25, 41)	< 0.0001
Year of the index date, median (IQR)	2010 (2007, 2013)	2010 (2007, 2013)	2010 (2007, 2012)	NS
Geographical region, *n* (%)				< 0.0001
London	158 (10.6)	92 (7.5)	21 (6.2)	
South England	406 (27.3)	300 (24.6)	92 (27.2)	
Midlands and East England	274 (18.4)	249 (20.4)	76 (22.5)	
North England	243 (16.3)	236 (19.3)	53 (15.7)	
Northern Ireland	53 (3.6)	92 (7.5)	23 (6.8)	
Scotland	189 (12.7)	125 (10.3)	42 (12.4)	
Wales	164 (11.0)	126 (10.3)	31 (9.2)	
Smoking, *n* (%)				0.022
Non-smoker	495 (33.3)	387 (31.7)	129 (38.2)	
Ever smoker	802 (53.9)	768 (63.0)	194 (57.4)	
Unknown	190 (12.8)	65 (5.3)	15 (4.4)	
Alcohol consumption, *n* (%)				0.005
Non-drinker	155 (10.4)	112 (9.2)	31 (9.2)	
Ever drinker	730 (49.1)	777 (63.7)	235 (69.5)	
Unknown	602 (40.5)	331 (27.1)	72 (21.3)	
Body mass index, median (IQR)	23.4 (20.9, 27.1)	23.8 (21.0, 28.1)	25.0 (21.3, 30.2)	< 0.0001
< 18.5 kg/m^2^ (underweight), *n* (%)	65 (4.4)	61 (5.0)	24 (7.1)	
≥ 18.5 kg/m^2^ and < 25 kg/m^2^ (normal), *n* (%)	502 (33.8)	460 (37.7)	123 (36.4)	
≥ 25 kg/m^2^ and < 30 kg/m^2^ (overweight), *n* (%)	215 (14.5)	215 (17.6)	71 (21.0)	
≥ 30 kg/m^2^ (obese), *n* (%)	125 (8.4)	154 (12.6)	76 (22.5)	
Unknown, *n* (%)	580 (39.0)	330 (27.1)	44 (13.0)	
Number of different prescriptions in 5 years before diagnosis, median (IQR)	3 (2, 5)	5 (3, 7)	7 (5, 8)	< 0.0001
Number of GP consultations in 5 years before diagnosis, median (IQR)	36 (19, 65)	66 (42, 103)	119 (79, 171)	< 0.0001
4–5 years prior to diagnosis	4 (1, 10)	8 (3, 17)	15 (8, 27)	< 0.0001
3–4 years prior to diagnosis	4 (1, 11)	9 (3, 18)	18 (9, 33)	< 0.0001
2–3 years prior to diagnosis	5 (1, 11)	10 (4, 20)	21 (11, 33)	< 0.0001
1–2 years prior to diagnosis	6 (2, 13)	13 (5, 22)	21 (12, 40)	< 0.0001
0–1 year prior to diagnosis	11 (5, 21)	21 (11, 33)	32 (19, 52)	< 0.0001

*FEP* first episode psychosis, *NMSC* no or minimal symptom cluster, *ASC* affective symptom cluster, *MSC* multiple symptom cluster, *NS* not significant

^†^Obtained from chi-squared or univariable multinomial logistic regression analysis as appropriate, and where applicable analysis excluded the unknown category (missing data)

^‡^Data were not reported for pregnancy-related psychosis due to CPRD reporting policy that no cell should contain fewer than five events

### Symptom clusters emerging over time before FEP diagnosis

**Fig. 2:**
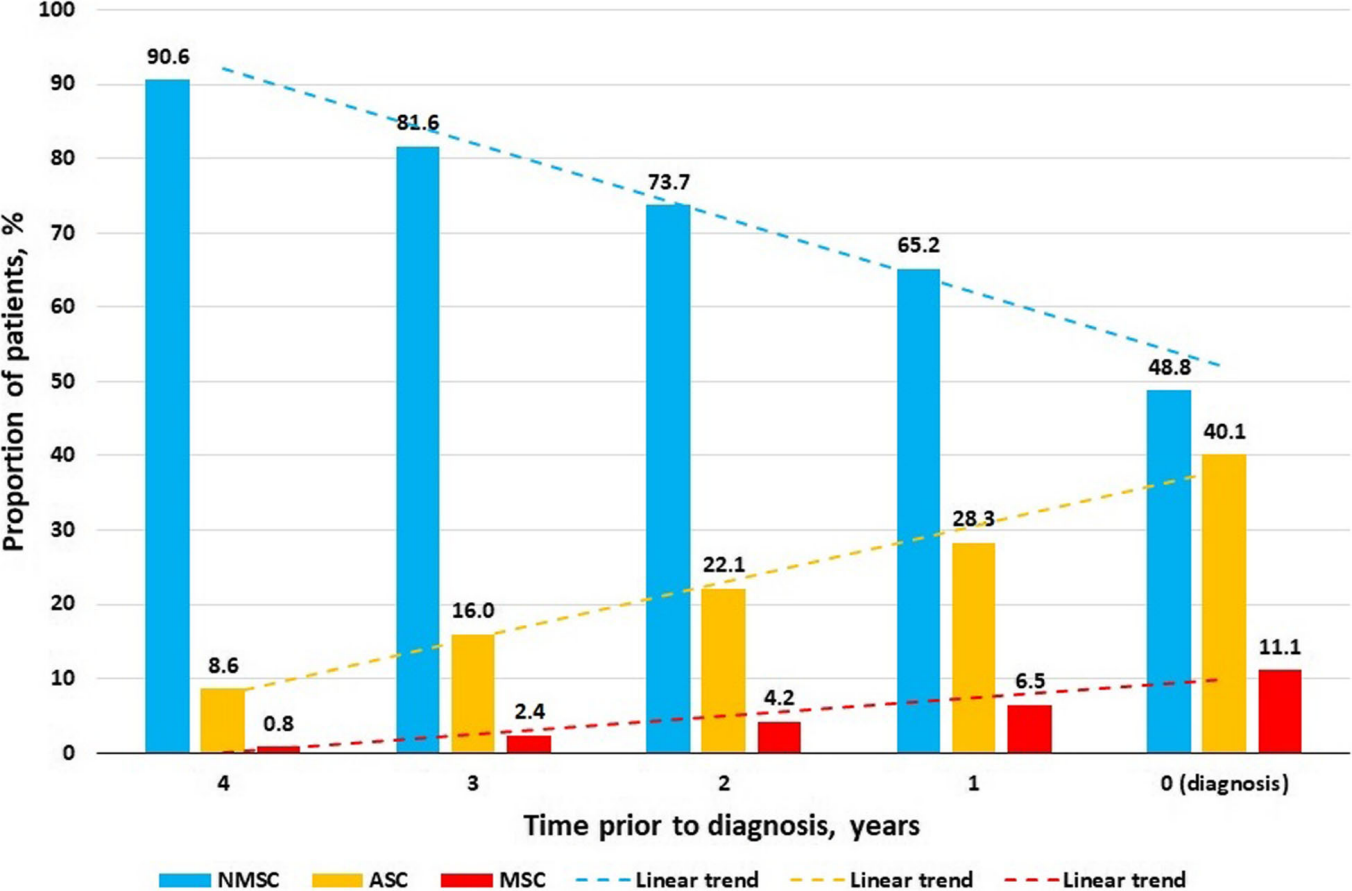
Proportion of patients allocated to the three clusters based on pre-recorded symptoms at different time points before FEP diagnosis. FEP, first episode psychosis; NMSC, no or minimal symptom cluster; ASC, affective symptom cluster; MSC, multiple symptom cluster. Symptom clusters are identified based on the data from 5 years before diagnosis up to each time point

Nine per cent of FEP patients could already be classified into the affective symptom cluster at 4 years before diagnosis and 28% at 1 year before diagnosis. One per cent of FEP patients could be classified in the multiple symptom cluster 4 years before diagnosis, rising to 7% at 1 year before diagnosis (Fig. 2).

### Discriminative ability of symptom clusters on FEP diagnosis

The majority of controls (87%, *n* = 10,554) were mapped to the no or minimal symptom cluster, followed by the affective (11%, *n* = 1314) and multiple (3%, *n* = 312) symptom clusters. The affective and multiple symptom clusters (versus the no or minimal symptom cluster) demonstrated a good discriminative ability for FEP predictive classification (C-statistic 0.766 (95% CI 0.757, 0.775); sensitivity = 51.2%; specificity = 86.7%).

## Discussion

This large study utilising a national primary care database has shown three distinct patterns of symptom presentation prior to FEP diagnosis, with more than one in ten patients presenting with multiple different symptoms in the 5 years prior to diagnosis. Many patients diagnosed with FEP have a long history of relevant symptom presentation to primary care.

### Major findings

This study has shown that symptoms are often presented several years prior to the diagnosis of FEP. The median time interval between the first recorded potential prodromal symptom and a coded FEP diagnosis was around two or more years. This is longer than the reported average DUP [2–4], although our study in primary care cannot assess whether the recorded symptoms are part of the FEP prodrome.

In particular, there are a group of patients (11% of all those diagnosed with FEP) who have presented with multiple symptoms in the previous 5 years before diagnosis. This group has high rates of consultations and present with a range of morbidity before the diagnosis of FEP. This group may represent patients who could be recognised sooner as the median time of the earliest recorded symptom is about 4 years before FEP. Approximately two thirds of this cluster were female, and it may be that GPs are less likely to recognise potential FEP in females. The second group of patients (40%) mainly presented with affective symptoms (such as mood-related and neurotic symptoms) prior to diagnosis. The third group of patients (49%) had no or minimal symptoms recorded in primary care, suggesting either an insidious onset disease or limited prior use of primary care. The group tended to be of younger age at diagnosis, male, with lower BMI, morbidity burden, and lower rate of GP consultations.

We found that many patients who later go on to receive the diagnosis of FEP consulted their GP with increasing regularity over the 5 years before diagnosis and particularly in the 12 months before diagnosis (more than quadruple the frequency of consultation than our control group). These findings confirm this population in general is actively help-seeking, but those prodromal symptom presentations may be difficult for GPs to elicit and distinguish from less severe states and disorders. However, there remains a quarter of patients with no record of coded symptoms in the primary care database, who may not be actively seeking related help from their GP in the 5 years before FEP diagnosis.

High rates of recorded physical co-morbidities such as respiratory conditions might be expected in a population known to smoke heavily [25]. The high rates of recorded physical symptoms, and with comorbid musculoskeletal pain, are a novel finding and may relate to the increased levels of somatic complaints prior to FEP diagnosis. Somatic complaints have previously been recognised to be a frequent manifestation of psychological distress in common mental health disorders such as depression and anxiety [26].

Mood-related, ‘neurotic’, and physical symptoms were among the most frequently recorded. Common psychological symptoms that characterise the at risk mental state or the ultrahigh risk stage before the actual diagnosis of psychosis include social isolation or withdrawal, impairment in personal hygiene and grooming, blunted or inappropriate affect, odd beliefs or magical thinking, and unusual perceptual experiences [27]. These were not commonly recorded symptoms in primary care, but once coded, heralded a shorter period to diagnosis. GPs may be reluctant to enquire about psychotic symptoms. It is possible that the low prevalence of these symptoms in the GP records is due to inadequate awareness that these clinical manifestations may herald the onset of psychosis.

Substance misuse was commonly recorded in FEP patients. This underlines the importance of GPs recognising that FEP is commonly preceded or accompanied by co-morbid substance misuse. It is therefore important not to misattribute relevant symptom presentations purely to substance misuse without careful exploration of the possibility of an emerging psychotic disorder [28].

The affective and multiple symptom cluster patterns were rare in our control group. Our discriminative ability analysis for FEP classification has shown that these two patterns of symptom presentation are suggestive (C-statistic 0.766) for a potential FEP. Importantly, many patients already had these symptom patterns several years before a FEP diagnosis (one third 1 year before diagnosis, a quarter at 2 years before diagnosis, a fifth at 3 years before diagnosis), giving an opportunity for earlier awareness, referral, and diagnosis.

### Comparison with the existing literature

The literature on the patterns of symptoms in the potentially long prodromal period before the onset of psychosis is limited. Most studies on the identification of symptom patterns focus on help-seeking ultrahigh-risk populations in specialised mental health service settings [29–31] or examined individual symptoms prior to the diagnosis [32]. We are not aware of any other previous study that has investigated the pattern of symptoms presentation for individuals who are help-seeking in primary care over a long observational period prior to diagnosis, although a survey investigation has modelled subtypes of psychosis-like experiences in the general population using similar latent-class analytical approach [33].

An American study described patterns of health care use before FEP in adolescents and young adults. Although it included previous diagnoses of depressive, anxiety, attention deficit, bipolar, and substance use disorders as an indication of mental health problem, the pattern of symptoms and their relationship with the diagnosis of FEP was not studied [34]. Sullivan et al. examined 13 individual symptoms recorded in primary care to assess the positive predictive value of single or paired symptoms for a diagnosis of psychosis between 2000 and 2009 [32]. The majority of the symptoms (*n* = 12) were linked to later psychosis diagnosis, with suicidal behaviour as the most strongly associated common predictor. We also found a strong association with suicidal behaviour and self-harm but have extended Sullivan’s study by examining 55 individual symptoms and determining common patterns of presentation prior to diagnosis.

### Strengths and limitations

The increasing availability and quality of routinely recorded longitudinal primary care electronic health records offer the opportunity to investigate the patterns of symptoms recorded before a FEP diagnosis in a larger and more generalizable setting. We restricted the analysis to the period following the introduction of the QOF in the UK which increased the quality of recording in primary care. However, the recording of diagnosis of FEP made in secondary care may be delayed in entry into primary care records, and patients with a diagnosis of psychosis recorded only in secondary care would not be included in our analysis. We excluded those with a long history of antipsychotic medication (more than 1 year) which should mean patients with FEP included in our analysis had a recent diagnosis, and this study used a UK national primary care database (CPRD) with previous validation of the accuracy and completion of diagnosis recording of mental and behavioural disorders [13]. The median age (30 years old) at FEP diagnosis in our study was comparable with that (31) at a treated incidence of psychotic disorder in a recent large European (including England) multinational study [35]. A 5-year observational period was considered long enough to identify insidious symptoms related to FEP, although it is possible that relevant symptoms will present more than 5 years before FEP diagnosis. Not all symptoms identified will necessarily have reflected a prodromal phase of FEP in all patients. A further limitation to this study is the lack of primary care recording of concerns raised by families, potentially an important factor in alerting a GP to the presence of a more serious mental disorder. We included over 50 individual symptoms identified through a review of the literature [17–19], but other symptoms may be missed. We summarised the psychological symptoms into 6 groups largely based on the work of Yung and McGorry [17], but there may be other approaches to grouping the symptoms. We included 15 physical symptoms together as a single group, but we are aware that these somatic complaints are diverse in nature. It is possible that symptoms may not be coded but are recorded in the consultation free text that GPs use alongside the Read codes. However, it is likely that those with a coded symptom are those with more troublesome or noticeable symptoms. No attempt was made to grade the degree of each symptom. There was some missing data on covariates including smoking, alcohol drinking, and BMI, as is usual in research using primary care health record databases and particularly in younger populations. The rapid development of the early intervention in psychosis services in England between 2000 and 2010 may have caused geographical variation in the responsiveness of GPs to psychosis presentations where they had direct access to specialist FEP assessment [36]. The generalisability of the findings from this study has yet to be assessed.

### Impact and implications

The criteria that use psychiatric and cognitive symptoms for recognition of prodromal stage or at risk mental state for psychosis do not work well outside defined clinical population samples and not within the general population seeking help in primary care [37]. This study highlights the opportunities in primary care for identifying patients who may be experiencing a prodromal state of psychosis rather than awaiting the emergence of major psychotic symptoms or acute psychosis. Our study has highlighted there is a significant minority (often female) who may be waiting several years for a diagnosis and are actively seeking help in primary care. This is a group whose characteristics GPs should be particularly be aware of to allow the opportunity for earlier recognition, referral, and diagnosis. The three clusters suggested in the present study represent the first attempt to link common prodromal presentations in primary care to the identification of subsequent FEP. The curriculum and training programmes for GPs need a greater focus on early detection of symptoms suggestive of psychosis and its prodrome. It may be possible to link these symptoms and clusters with existing criteria and other markers (such as increasing frequency of GP attendance, suicidality, social withdrawal, and a history of severe mental illness) to identify the people at high risk of psychosis at the earliest possible stage, as a risk prediction model, hence contributing to more effective treatment strategies to improve outcomes.

### Patient and public involvement

The findings were discussed with a convenor of a local support group (‘Hear Our Voices’, North Staffordshire, England) that aims to give a voice to people with a mental health problem. He suggested that the findings made sense and emphasised the reluctance of men, who might use alcohol and cigarettes as a coping strategy, to access care. He suggested that the association of lower BMI with FEP patients may reflect a lack of self-care despite possible metabolic abnormalities, and the observation of increased injury and major trauma in FEP patients may be due to excess risky behaviours. He was concerned that suicidality was recorded in about 6% of people who were later diagnosed with FEP, about 14 times commoner than in controls. He emphasised the importance of this work in providing clues for GPs to increase their awareness of the possibility of an emerging psychosis.

## Conclusions

Our study identified three distinctive patterns of prodromal symptom presentations in patients seeking help in primary care and subsequently diagnosed with FEP. Awareness of these symptom clusters may help GPs to identify patients who are experiencing a prodromal state of psychosis, thereby facilitating more timely access to specialist assessment and treatment and hence better long-term outcomes.

## Supplementary information

**Additional file 1.** Comparison of patient characteristics and patterns of symptoms in FEP patients with and without missing data on smoking, alcohol drinking and BMI.

**Additional file 2.** Definitions (psychosis, prodromal symptom and antipsychotic medication).

**Additional file 3.** Comorbid conditions measured at index date in FEP patients and matched participants.

**Additional file 4.** Five-year prevalence of prodrome symptoms in FEP patients and matched participants.

**Additional file 5.** Time interval between first symptom (if any) and FEP diagnosis within each symptom group.

**Additional file 6.** Profile of the earliest symptom, among all symptom groups, in patients with FEP.

**Additional file 7.** Statistical assessment of the optimal number of clusters from latent class analysis models based on eight groups of prodrome symptoms.

## Data Availability

No additional data or material available if not stated elsewhere.

## Abbreviations

BIC: Bayes Information Criterion
BMI: Body mass index
BNF: British National Formulary
CAIC: Consistent Akaike’s Information Criterion
CI: Confidence interval
CPRD: Clinical Practice Research Datalink
DUP: Duration of untreated psychosis
FEP: First episode psychosis
GP: General practitioner
LCA: Latent class analysis
OR: Odds ratio
QOF: Quality and Outcomes Framework
