# Supplementary material for: Patterns of symptoms before a diagnosis of first episode psychosis: a latent class analysis of UK primary care electronic health records
Source: BMC Med. 2019 Dec 4;17:227. doi: 10.1186/s12916-019-1462-y (PMC6894287; doi:10.1186/s12916-019-1462-y)
Supplement: Supplementary file 1 — Additional file 1. Comparison of patient characteristics and patterns of symptoms in FEP patients with and without missing data on smoking, alcohol drinking and BMI. [file 12916_2019_1462_MOESM1_ESM.docx]

**Demographic and clinical characteristics at diagnosis in FEP patients with and without missing data on smoking, alcohol drinking and BMI.**

|  | | | **FEP patients with missing data (*n*=1,356)** | **FEP patients without missing data (*n*=1,689)** |
| --- | --- | --- | --- | --- |
| Type of psychosis, *n* (%) | | |  |  |
|  | Non-affective | | 935 (69.0) | 1,101 (65.2) |
|  | Drug-induced | | 303 (22.4) | 375 (22.2) |
|  | Affective | | 114 (8.4) | 195 (11.6) |
|  | Pregnancy-related* | | / | 18 (1.1) |
| Year of index date, median (IQR) | | | 2010 (2007, 2013) | 2010 (2007, 2013) |
| Age, median (IQR) | | | 24 (20, 32) | 35 (28, 41) |
| Male, *n* (%) | | | 1,001 (73.8) | 913 (54.1) |
| Geographical region, *n* (%) | | |  |  |
|  | London | | 119 (8.8) | 152 (9.0) |
|  | South, England | | 405 (29.9) | 393 (23.3) |
|  | Midlands and East, England | | 256 (18.9) | 343 (20.3) |
|  | North, England | | 204 (15.0) | 328 (19.4) |
|  | Northern Ireland | | 66 (4.9) | 102 (6.0) |
|  | Scotland | | 161 (11.9) | 195 (11.6) |
|  | Wales | | 145 (10.7) | 176 (10.4) |
| Smoking, *n* (%) | | |  |  |
|  | Non smoker | | 421 (31.1) | 590 (34.9) |
|  | Ever smoker | | 665 (49.0) | 1,099 (65.1) |
|  | Unknown | | 270 (19.9) | / |
| Alcohol consumption, *n* (%) | | |  |  |
|  | Non drinker | | 74 (5.5) | 224 (13.3) |
|  | Ever drinker | | 277 (20.4) | 1,465 (86.7) |
|  | Unknown | | 1,005 (74.1) | / |
| Body mass index, median (IQR) | | | 22.8 (20.1, 26.8) | 24.0 (21.2, 28.2) |
|  | < 18.5 kg/m^2^ (underweight), *n* (%) | | 52 (3.8) | 98 (5.8) |
|  | ≥ 18.5 kg/m^2^ & < 25 kg/m^2^ (normal), *n* (%) | | 215 (15.9) | 870 (51.5) |
|  | ≥ 25 kg/m^2^ & < 30 kg/m^2^ (overweight), *n* (%) | | 79 (5.8) | 422 (25.0) |
|  | ≥ 30 kg/m^2^ (obese), *n* (%) | | 56 (4.1) | 299 (17.7) |
|  | Unknown | | 954 (70.4) | / |
| Number of different prescriptions in 5 years before index date, median (IQR) | | | 4 (2, 5) | 5 (3, 7) |
| Number of GP consultations in 5 years before index date, median (IQR) | | | 40 (21, 69) | 69 (39, 116) |
|  | | 4-5 year prior to index date | 4 (1, 10)† | 9 (3, 19)† |
|  | | 3-4 year prior to index date | 4 (1, 11)† | 10 (3, 21)† |
|  | | 2-3 year prior to index date | 5 (1, 12)† | 11 (4, 22)† |
|  | | 1-2 year prior to index date | 7 (2, 15)† | 12 (5, 24)† |
|  | | 0-1 year prior to index date | 13 (6, 24)† | 20 (10, 36)† |
| Number of symptom records in 5 years before index date, median (IQR)‡ | | | 1 (0, 4) | 3 (1, 6) |

FEP, first episode psychosis; BMI, body mass index; IQR, interquartile range. †One-way ANOVA trend analysis within patients with FEP, p < 0.0001. ‡Total number of coded records based on 55 studied individual symptoms. *Data were not reported for certain cells due to CPRD reporting policy that no cell should contain fewer than 5 events.

**Cumulative proportion of patients having a prodrome symptom recorded in primary care from 5 years before until the time of FEP diagnosis in patients with missing data (smoking, alcohol drinking or BMI).**

**
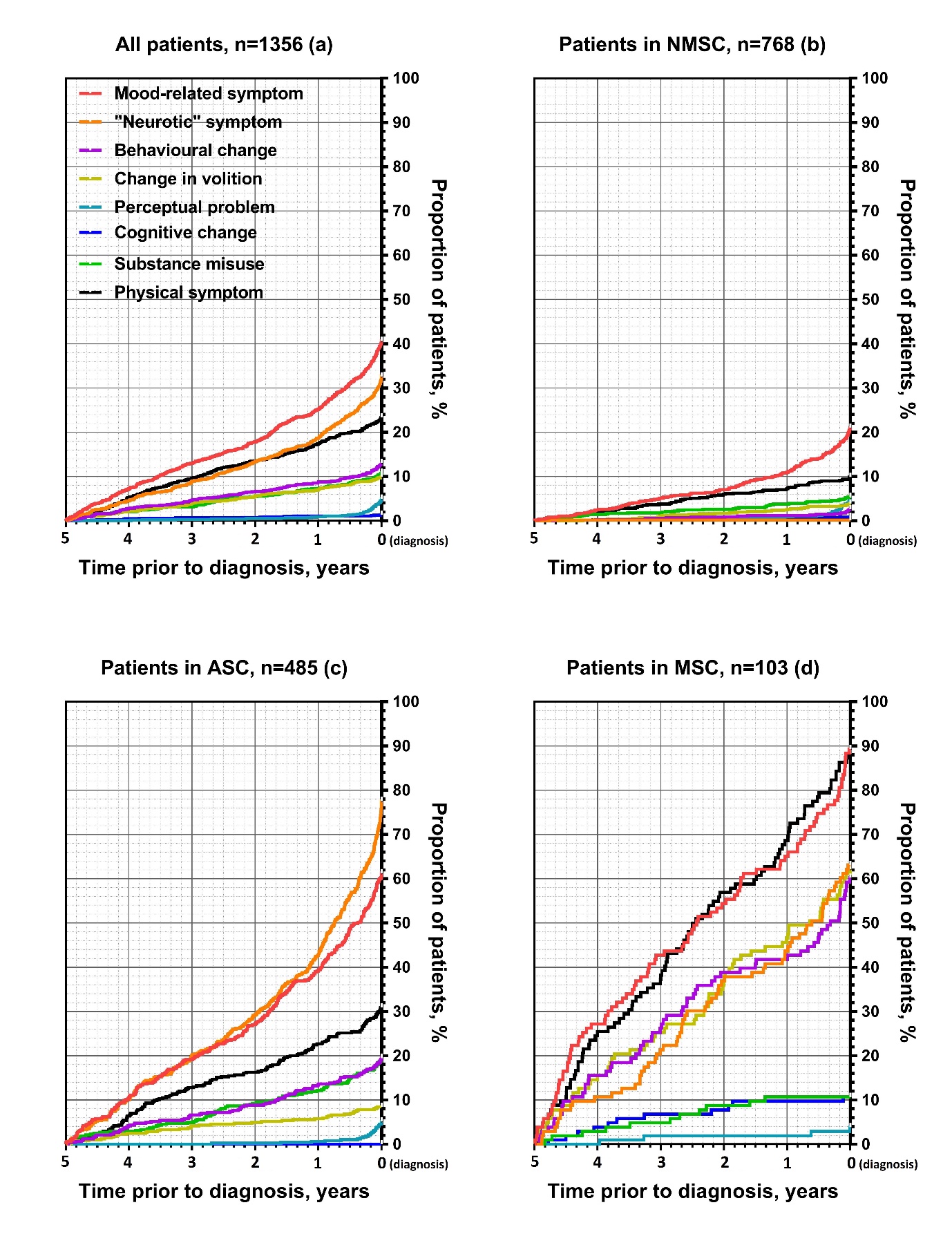
**

**Cumulative proportion of patients having a prodrome symptom recorded in primary care from 5 years before until the time of FEP diagnosis in patients without missing data (smoking, alcohol drinking and BMI).**

**
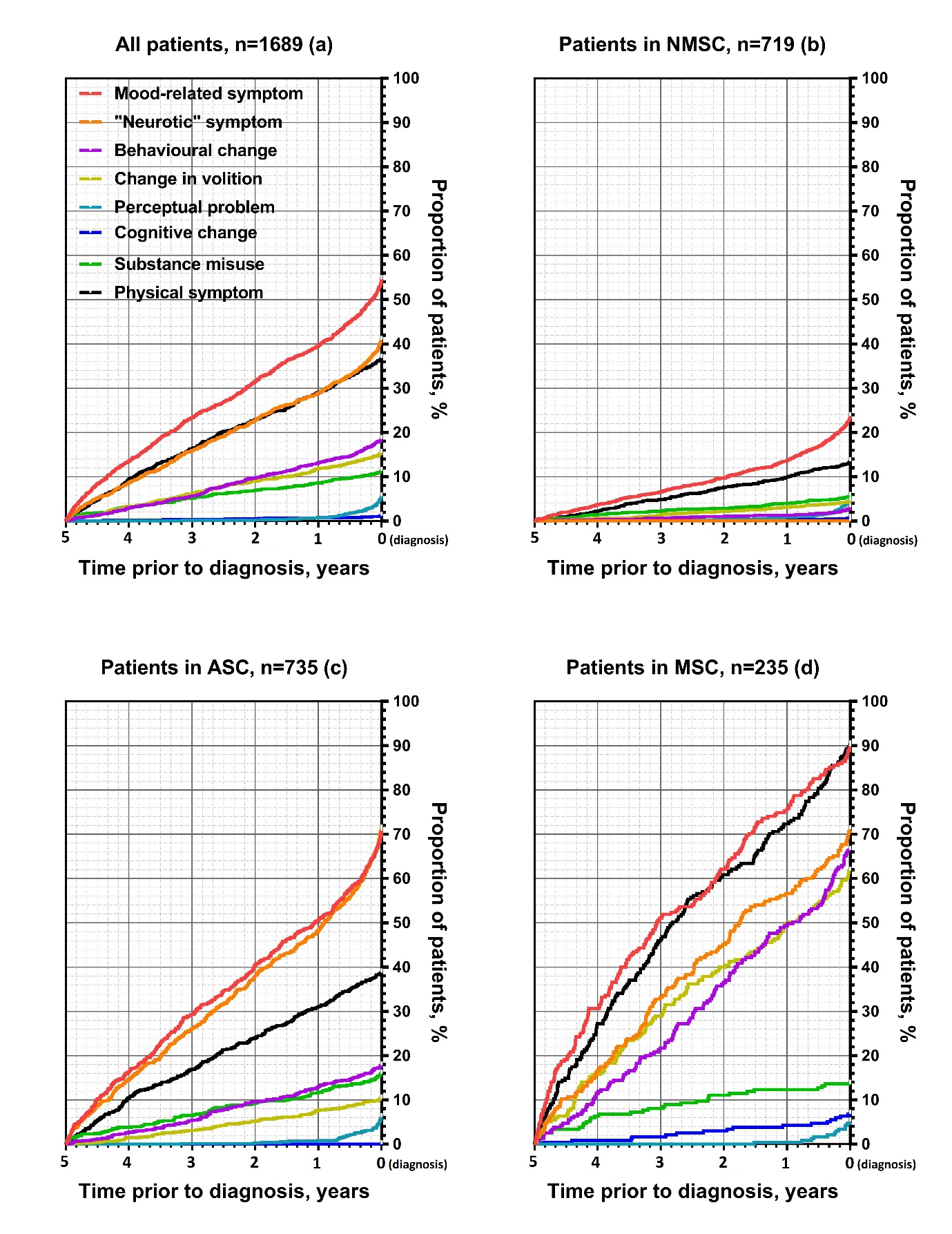
**
