# Supplementary material for: Patterns of symptoms before a diagnosis of first episode psychosis: a latent class analysis of UK primary care electronic health records
Source: BMC Med. 2019 Dec 4;17:227. doi: 10.1186/s12916-019-1462-y (PMC6894287; doi:10.1186/s12916-019-1462-y)
Supplement: Supplementary file 3 — Additional file 3. Comorbid conditions measured at index date in FEP patients and matched participants. [file 12916_2019_1462_MOESM3_ESM.docx]

**Comorbid conditions measured at index date in FEP patients and matched participants**

| **Comorbid condition** | | | **FEP patients**  **(*n*=3,045)** | **Matched participants**  **(*n*=12,180)** | ***p*-value‡** |
| --- | --- | --- | --- | --- | --- |
| Respiratory condition (any), *n* (%) | | | 337 (11.1) | 1,077 (8.84) | < 0.0001 |
|  | | Asthma | 333 (10.9) | 1,067 (8.76) | < 0.0001 |
|  | | COPD | 9 (0.30) | 25 (0.21) | NS |
| Inflammatory condition (any), *n* (%) | | | 31 (1.02) | 134 (1.10) | NS |
|  | Rheumatoid arthritis* | | / | 6 (0.05) | / |
|  | Gout | | 15 (0.49) | 59 (0.48) | NS |
|  | Polymyalgia rheumatic* | | / | / | / |
|  | Inflammatory bowel disease | | 9 (0.30) | 51 (0.42) | NS |
|  | Systemic lupus erythematosus* | | / | / | / |
|  | Spondyloarthritis† | | 6 (0.20) | 17 (0.14) | NS |
| Hypertension, *n* (%) | | | 67 (2.20) | 247 (2.03) | NS |
| IHD, *n* (%) | | | 7 (0.23) | 13 (0.11) | NS |
| Diabetes, *n* (%) | | | 52 (1.71) | 162 (1.33) | NS |
| Chronic kidney disease, *n* (%) | | | 10 (0.33) | 27 (0.22) | NS |
| Musculoskeletal pain (any), *n* (%) | | | 1,218 (40.0) | 3,872 (31.8) | < 0.0001 |
|  | | Back | 559 (18.4) | 1,553 (12.8) | < 0.0001 |
|  | | Foot/ankle | 279 (9.16) | 985 (8.09) | NS |
|  | | Hand/wrist | 297 (9.75) | 854 (7.01) | < 0.0001 |
|  | | Hip | 5 (0.16) | 20 (0.16) | NS |
|  | | Knee | 264 (8.67) | 895 (7.35) | 0.014 |
|  | | Neck | 239 (7.85) | 668 (5.48) | < 0.0001 |
|  | | Shoulder | 111 (3.65) | 342 (2.81) | 0.015 |
| Injury and major trauma, *n* (%) | | | 353 (11.6) | 1,043 (8.56) | < 0.0001 |

FEP, first episode psychosis. †Including ankylosing spondylitis and psoriatic arthritis; ‡Obtained from Chi-square; *Data were not reported for certain cells due to CPRD reporting policy that no cell should contain fewer than 5 events. NS, not significant.
