# Supplementary material for: Patterns of symptoms before a diagnosis of first episode psychosis: a latent class analysis of UK primary care electronic health records
Source: BMC Med. 2019 Dec 4;17:227. doi: 10.1186/s12916-019-1462-y (PMC6894287; doi:10.1186/s12916-019-1462-y)
Supplement: Supplementary file 4 — Additional file 4. Five-year prevalence of prodrome symptoms in FEP patients and matched participants. [file 12916_2019_1462_MOESM4_ESM.docx]

**Five-year prevalence of prodrome symptoms in FEP patients and matched participants**

|  |  | **FEP Patients**  **(*n*=3,045)** | | **Matched participants**  **(*n*=12,180)** | | **Odds ratio (95% CI)†** |
| --- | --- | --- | --- | --- | --- | --- |
| **Symptom group**  **Individual symptom** | | ***n*** | **5-year prevalence in %** | ***n*** | **5-year prevalence in %** |  |
| Mood-related symptom, any | | 1,473 | 48.37 | 1,275 | 10.47 | 10.1 (9.12, 11.3) |
|  | Depression | 1,394 | 45.78 | 1,228 | 10.08 | 9.37 (8.45, 10.4) |
|  | Suicidal/self-harm ideas or attempts | 189 | 6.21 | 62 | 0.51 | 13.5 (10.0, 18.0) |
|  | Mood swings | 60 | 1.97 | 54 | 0.44 | 4.65 (3.20, 6.74) |
|  | Anhedonia* | / | / | / | / | / |
|  | Guilt* | / | / | 5 | 0.04 | / |
| ‘Neurotic’ symptom, any | | 1,133 | 37.21 | 1,106 | 9.08 | 6.54 (5.89, 7.27) |
|  | Anxiety | 879 | 28.87 | 791 | 6.49 | 6.48 (5.79, 7.26) |
|  | Neuroses | 249 | 8.18 | 227 | 1.86 | 4.97 (4.09, 6.05) |
|  | Irritability and anger | 97 | 3.19 | 91 | 0.75 | 4.40 (3.34, 5.80) |
|  | Restlessness | 107 | 3.51 | 21 | 0.17 | 24.6 (13.7, 44.3) |
|  | Worrying thoughts | 58 | 1.90 | 93 | 0.76 | 2.77 (2.01, 3.82) |
| Behavioural change, any | | 490 | 16.09 | 657 | 5.39 | 3.53 (3.08, 4.05) |
|  | Deterioration | 387 | 12.71 | 565 | 4.64 | 3.16 (2.71, 3.61) |
|  | Aggressive and disruptive behaviour | 101 | 3.32 | 77 | 0.63 | 5.52 (4.10, 7.45) |
|  | Odd behaviour | 19 | 0.63 | 11 | 0.09 | / |
|  | Reduced self-esteem | 12 | 0.39 | 14 | 0.11 | / |
|  | Social withdrawal* | / | / | / | / | / |
|  | Impulsivity* | / | / | / | / | / |
| Change in volition, any | | 394 | 12.94 | 923 | 7.58 | 1.88 (1.64, 2.16) |
|  | Tiredness/fatigue (loss of energy) | 393 | 12.91 | 911 | 7.48 | 1.90 (1.66, 2.18) |
|  | Boredom (loss of interest)* | / | / | 12 | 0.10 | / |
|  | Apathy (loss of drive)* | / | / | 9 | 0.07 | / |
| Perceptual problem, any | | 162 | 5.32 | 5 | 0.04 | 130 (53.7, 313) |
|  | Hallucinations* | 93 | 3.05 | / | / | / |
|  | Delusions* | 73 | 2.40 | / | / | / |
|  | Illusions* | / | / | / | / | / |
| Cognitive change, any | | 38 | 1.25 | 30 | 0.25 | 5.41 (3.35, 8.75) |
|  | Concentration/preoccupation difficulties | 16 | 0.53 | 18 | 0.15 | / |
|  | Cognitive/memory impairment | 16 | 0.53 | 9 | 0.07 | / |
|  | Thought disorder/blocking* | 5 | 0.16 | / | / | / |
|  | Disturbance of attention* | / | / | / | / | / |
| Substance misuse, any | | 338 | 11.10 | 134 | 1.10 | 13.0 (10.4, 16.4) |
|  | General (codes without specific substance) | 191 | 6.27 | 61 | 0.50 | 15.9 (11.4, 22.1) |
|  | Opioids | 68 | 2.23 | 24 | 0.20 | 12.2 (7.28, 20.5) |
|  | Alcohol | 56 | 1.84 | 35 | 0.29 | 6.81 (4.41, 10.5) |
|  | Cannabis | 65 | 2.13 | 16 | 0.13 | 16.3 (9.32, 28.3) |
|  | Hypnotic | 33 | 1.08 | 10 | 0.08 | 14.4 (6.73, 30.9) |
|  | Cocaine | 16 | 0.53 | 7 | 0.06 | / |
|  | Amphetamine* | 11 | 0.36 | / | / | / |
|  | Glue* | / | / | / | / | / |
|  | Other/multiple stimulant* | / | / | / | / | / |
|  | Tobacco* | / | / | / | / | / |
|  | Hallucinogen* | / | / | / | / | / |
|  | Ecstasy* | / | / | / | / | / |
|  | Antidepressant* | / | / | / | / | / |
|  | Solvent* | / | / | / | / | / |
| Physical symptom, any | | 939 | 30.84 | 1,926 | 15.81 | 2.70 (2.43, 2.99) |
|  | Sleep disturbance | 399 | 13.10 | 338 | 2.78 | 5.51 (4.64, 6.54) |
|  | Menstrual problem – female‡ | 325 | 28.74 | 910 | 20.11 | 1.63 (1.38, 1.92) |
|  | Shortness of breath | 126 | 4.14 | 250 | 2.05 | 2.08 (1.69, 2.57) |
|  | Epigastric discomfort | 95 | 3.12 | 236 | 1.94 | 1.64 (1.31, 2.05) |
|  | Palpitations | 77 | 2.53 | 221 | 1.81 | 1.40 (1.08, 1.82) |
|  | Excessive wind | 53 | 1.74 | 114 | 0.94 | 1.92 (1.34, 2.76) |
|  | Loss of weight | 41 | 1.35 | 69 | 0.57 | 2.43 (1.65, 3.58) |
|  | Poor appetite | 25 | 0.82 | 44 | 0.36 | / |
|  | Failure of erection - male‡ | 21 | 1.10 | 32 | 0.42 | 2.66 (1.52, 4.65) |
|  | Dysphagia | 20 | 0.66 | 32 | 0.26 | / |
|  | Muscle tension | 11 | 0.36 | 7 | 0.06 | / |
|  | Decreased libido | 13 | 0.43 | 32 | 0.26 | / |
|  | Hyperventilation | 7 | 0.23 | 24 | 0.20 | / |
|  | Speech abnormalities* | 5 | 0.16 | / | / | / |
|  | Dryness of mouth | 5 | 0.16 | 15 | 0.12 | / |

FEP, first episode psychosis; CI, confidence interval; †Obtained from conditional logistic regression analyses with cluster-robust variance estimator, unadjusted, and only analysed where a 5-year prevalence ≥ 1% in FEP; ‡In subgroup of particular gender. *Data were not reported for certain cells due to CPRD reporting policy that no cell should contain fewer than 5 events.
