# Supplementary material for: Patterns of symptoms before a diagnosis of first episode psychosis: a latent class analysis of UK primary care electronic health records
Source: BMC Med. 2019 Dec 4;17:227. doi: 10.1186/s12916-019-1462-y (PMC6894287; doi:10.1186/s12916-019-1462-y)
Supplement: Supplementary file 5 — Additional file 5. Time interval between first symptom (if any) and FEP diagnosis within each symptom group. [file 12916_2019_1462_MOESM5_ESM.docx]

**Time interval between first symptom (if any) and FEP diagnosis within each symptom group**

|  | **All FEP patients**  **(*n*=3,045)** | | **Patients in NMSC**  **(*n*=1,487)** | | **Patients in ASC (*n*=1,220)** | | **Patients in MSC**  **(*n*=338)** | |
| --- | --- | --- | --- | --- | --- | --- | --- | --- |
| **Symptom group** | ***n*† (%)** | **Time interval, median days (IQR)** | ***n*† (%)** | **Time interval, median days (IQR)** | ***n*† (%)** | **Time interval, median days (IQR)** | ***n*† (%)** | **Time interval, median days (IQR)** |
| Mood-related symptom | 1,473 (48.4) | 784 (260, 1399) | 352 (23.7) | 538 (148, 1192) | 818 (67.0) | 777 (260, 1374) | 303 (89.6) | 1162 (585, 1591) |
| ‘Neurotic’ symptom‡ | 1,133 (37.2) | 732 (223, 1297) | / | / | 900 (73.8) | 652 (199, 1271) | 232 (68.6) | 970 (400, 1375) |
| Behavioural change | 490 (16.1) | 784 (238, 1247) | 46 (3.1) | 221 (42, 1019) | 226 (18.5) | 738 (271, 1255) | 218 (64.5) | 868 (291, 1313) |
| Change in volition | 394 (12.9) | 895 (365, 1364) | 68 (4.6) | 677 (264, 1122) | 117 (9.6) | 802 (316, 1316) | 209 (61.8) | 1015 (410, 1462) |
| Perceptual problem | 162 (5.3) | 70 (20, 213) | 76 (5.1) | 68 (20, 196) | 70 (5.7) | 60 (17, 213) | 16 (4.7) | 112 (29, 255) |
| Cognitive change‡ | 38 (1.3) | 758 (133, 1400) | 11 (0.7) | 508 (16, 1490) | / | / | 27 (8.0) | 850 (226, 1400) |
| Substance misuse | 338 (11.1) | 917 (321, 1421) | 84 (5.6) | 775 (298, 1483) | 211 (17.3) | 890 (291, 1314) | 43 (12.7) | 1271 (823, 1567) |
| Physical symptom | 939 (30.8) | 975 (402, 1456) | 201 (13.5) | 825 (344, 1323) | 435 (35.7) | 960 (421, 1456) | 303 (89.6) | 1072 (477, 1511) |

FEP, first episode psychosis; NMSC, no or minimal symptom cluster; ASC, affective symptom cluster; MSC, multiple symptom cluster; †Number of individuals ever recorded with studied symptom in the 5-year period before diagnosis; ‡Data were not reported for certain cells due to CPRD reporting policy that no cell should contain fewer than 5 events. IQR, interquartile range.
