# Supplementary material for: Patterns of symptoms before a diagnosis of first episode psychosis: a latent class analysis of UK primary care electronic health records
Source: BMC Med. 2019 Dec 4;17:227. doi: 10.1186/s12916-019-1462-y (PMC6894287; doi:10.1186/s12916-019-1462-y)
Supplement: Supplementary file 6 — Additional file 6. Profile of the earliest symptom, among all symptom groups, in patients with FEP. [file 12916_2019_1462_MOESM6_ESM.docx]

**Profile of the earliest symptom, among all symptom groups, in patients with FEP**

|  |  | **All FEP patients**  **(*n*=3,045)** | | **Patients in NMSC**  **(*n*=1,487)** | | | **Patients in ASC**  **(*n*=1,220)** | | | **Patients in MSC**  **(*n*=338)** | |
| --- | --- | --- | --- | --- | --- | --- | --- | --- | --- | --- | --- |
| **Symptom group (recorded as the earliest among studied groups)** | | ***n*† (%)** | **Time interval‡,**  **median days (IQR)** | ***n*† (%)** | **Time interval‡, median days (IQR)** | ***n*† (%)** | | **Time interval‡, median days (IQR)** | ***n*† (%)** | | **Time interval‡, median days (IQR)** |
| Any | | 2,311 (75.9) | 1065 (398, 1546) | 753 (50.6) | 600 (161, 1235) | 1,220 (100) | | 1120 (500, 1548) | 338 (100) | | 1548 (1193, 1729) |
|  | Mood-related symptom | 843 (27.7) | 1057 (348, 1560) | 347 (23.3) | 540 (151, 1213) | 391 (32.0) | | 1190 (640, 1603) | 105 (31.1) | | 1591 (1297, 1749) |
|  | ‘Neurotic’ symptom* | 578 (19.0) | 908 (291, 1524) | / | / | 524 (43.0) | | 850 (236, 1456) | 53 (15.7) | | 1580 (1182, 1724) |
|  | Behavioural change | 214 (7.0) | 1000 (399, 1509) | 46 (3.1) | 221 (42, 1019) | 110 (9.0) | | 992 (472, 1486) | 58 (17.2) | | 1383 (900, 1667) |
|  | Change in volition | 191 (6.3) | 1127 (505, 1563) | 59 (4.0) | 765 (265, 1163) | 67 (5.5) | | 1100 (387, 1484) | 65 (19.2) | | 1462 (1127, 1727) |
|  | Perceptual problem* | 51 (1.7) | 57 (20, 195) | 43 (2.9) | 57 (19, 189) | 6 (0.5) | | 46 (34, 107) | / | | / |
|  | Cognitive change* | 16 (0.5) | 1152 (104, 1643) | 10 (0.7) | 328 (16, 1490) | / | | / | 6 (1.8) | | 1592 (1400, 1746) |
|  | Substance misuse | 163 (5.4) | 1197 (532, 1704) | 69 (4.6) | 963 (355, 1539) | 82 (6.7) | | 1254 (890, 1753) | 12 (3.6) | | 1746 (1526, 1790) |
|  | Physical symptom | 495 (16.3) | 1230 (663, 1554) | 182 (12.2) | 825 (353, 1308) | 214 (17.5) | | 1314 (836, 1548) | 99 (29.3) | | 1549 (1237, 1735) |

FEP, first episode psychosis; NMSC, no or minimal symptom cluster; ASC, affective symptom cluster; MSC, multiple symptom cluster; †Number of individuals with the symptom as the earliest symptom in the 5-year period before diagnosis; *Data were not reported for certain cells due to CPRD reporting policy that no cell should contain fewer than 5 events. % may exceed from 100% due to multiple symptoms recorded on the same day, ‡Time interval between symptom (if any) and FEP diagnosis; IQR, interquartile range.
