# Supplementary material for: Patterns of symptoms before a diagnosis of first episode psychosis: a latent class analysis of UK primary care electronic health records
Source: BMC Med. 2019 Dec 4;17:227. doi: 10.1186/s12916-019-1462-y (PMC6894287; doi:10.1186/s12916-019-1462-y)
Supplement: Supplementary file 7 — Additional file 7. Statistical assessment of the optimal number of clusters from latent class analysis models based on eight groups of prodrome symptoms. [file 12916_2019_1462_MOESM7_ESM.docx]

**Statistical assessment of the optimal number of clusters from latent class analysis models based on eight groups of prodrome symptoms**

| Model | L^2^ statistics | Bootstrap *p* value | % reduction in L^2^ from H_0_ | Number of parameters | BIC_L_^2^ | CAIC_L_^2^ |
| --- | --- | --- | --- | --- | --- | --- |
| 1 cluster (H_0_) | 899.6 | < 0.0001 | - | 8 | -1081.7 | -1328.7 |
| 2 cluster | 197.0 | 0.028 | 78.1 | 17 | -1680.8 | -1909.8 |
| 3 cluster† | 156.0 | 0.43 | 82.7 | 26 | -1712.0 | -1950.0 |
| 4 cluster | 123.9 | 0.87 | 86.2 | 35 | -1640.8 | -1860.8 |
| 5 cluster | 107.7 | 0.94 | 88.0 | 44 | -1584.8 | -1795.8 |
| 6 cluster | 92.2 | 0.96 | 89.8 | 53 | -1528.1 | -1730.1 |

BIC_L_^2^, Bayes Information Criterion based on L-squared statistics; CAIC_L_^2^, Consistent Akaike’s Information Criterion based on L-squared statistics. †The optimal model.
